# Prospective clinical study of R-CMD therapy for indolent B cell lymphoma and mantle cell lymphoma from the Hokuriku Hematology Oncology Study Group

**DOI:** 10.1007/s12032-015-0677-9

**Published:** 2015-08-15

**Authors:** Tomoyuki Sakai, Yasufumi Masaki, Nozomi Otsuki, Ippei Sakamaki, Shinji Kishi, Takayoshi Miyazono, Yoshimasa Urasaki, Jun Murakami, Tomomi Satoh, Takuji Nakamura, Haruka Iwao, Akio Nakajima, Takafumi Kawanami, Miyuki Miki, Yoshimasa Fujita, Masao Tanaka, Toshihiro Fukushima, Toshiro Okazaki, Takanori Ueda

**Affiliations:** Department of Hematology and Immunology, Kanazawa Medical University, 1-1 Daigaku, Uchinada-machi, Kahoku-gun, Ishikawa 920-0293 Japan; Division of Clinical Research, National Organization Awara Hospital, 238-1 Kitagata, Awara, Fukui 910-4272 Japan; Division of Hematology and Oncology, Faculty of Medical Sciences, University of Fukui, 23-3 Matsuokashimoaizuki, Eiheijicho, Yoshidagun, Fukui 910-1193 Japan; Department of Gastroenterology and Hematology, University of Toyama, 2630 Sugitani, Toyama, Toyama 930-0194 Japan; Division of Transfusion Medicine, Faculty of Medical Sciences, University of Fukui, 23-3 Matsuokashimoaizuki, Eiheijicho, Yoshidagun, Fukui 910-1193 Japan

**Keywords:** Indolent B cell lymphoma, Mantle cell lymphoma, R-CMD, Toxicity

## Abstract

Standardized treatments for indolent B cell lymphoma primarily consisting of follicular lymphoma (FL) and for mantle cell lymphoma (MCL) have yet to be established. Here the Hokuriku Hematology Oncology Study Group conducted a multicenter prospective study to investigate the efficacy and safety of a combination regimen of rituximab, cladribine, mitoxantrone, and dexamethasone (R-CMD) in indolent B cell lymphoma and MCL. A total of 33 CD20-positive patients who received care between January 2008 and August 2011 were investigated. These patients’ illnesses were FL (*n* = 21), nodal marginal zone B cell lymphoma (NMZB, *n* = 3), MCL (*n* = 3), splenic marginal zone B cell lymphoma (*n* = 2), hairy cell leukemia (*n* = 1), Waldenstrom macroglobulinemia (WM, *n* = 1), and lymphoplasmacytic lymphoma (LPL, *n* = 2). Patients received four 21-day cycles of rituximab 375 mg/m^2^ (day 1), cladribine 0.10 mg/kg (days 1–3), mitoxantrone 8 mg/m^2^ (day 1), and dexamethasone 8 mg/body (days 1–3), with four additional rituximab doses at 4-week intervals. Of the 33 patients, 26 achieved complete response/unconfirmed complete response, and six achieved a partial response (4 with FL, 1 with NMZB, 1 with WM). One had progressive disease (FL), and four relapsed after remission (1 with FL, 2 with MCL, 1 with LPL). R-CMD therapy was relatively convenient and effective in indolent B cell lymphoma and MCL. Nonetheless, to suppress the number and function of both B cells and T cells, comprehensive infection prevention and follow-up are necessary in the future.

## Introduction

Standardized treatments have yet to be established for indolent B cell lymphoma, which predominantly consists of follicular lymphoma (FL). Although a temporary effect is observed with the conventional CHOP (cyclophosphamide, hydroxydaunorubicin, vincristine, and prednisolone) regimen, this regimen does not achieve curein indolent B cell lymphoma, and rather, indolent B cell lymphoma is known to have a worse long-term prognosis than aggressive lymphoma. In addition, standardized treatments have yet to be established for mantle cell lymphoma (MCL), a disease with poor prognosis that most commonly affects the elderly. Purine nucleoside derivatives such as fludarabine and cladribine block the adenosine metabolic pathway where adenosine deaminase is involved, and are known to be effective in indolent B cell lymphoma due to their treatment effects on cells in the quiescent state. The FMD regimen (fludarabine, mitoxantrone, and dexamethasone) [1–4] has been studied relatively frequently in recent years and has been shown to result in favorable treatment outcomes in indolent B cell lymphoma. However, currently in Japan, there is limited health insurance coverage for fludarabine injections, and using them for treating lymphoma is problematic at the present time. Because the mechanism of action and effects of fludarabine and cladribine are considered to be nearly equivalent, the CMD regimen, which is the FMD regimen but with fludarabine changed to cladribine, is considered to have nearly equivalent treatment effects as FMD. For this reason, the effects of CMD [5] and treatments that are similar to CMD [6–9] have been studied. Furthermore, rituximab is known to have a long blood half-life in in vivo kinetics, and its effects are sustained for a long time; thus, by adding rituximab to CMD (R-CMD), an enhancement in treatment effect can be anticipated. In a pilot study in our department, we confirmed the treatment effect and safety in patients who received R-CMD therapy. We therefore considered that it is necessary to verify its efficacy and safety in a phase II trial. Here, we report the results from this multicenter prospective clinical study.

## Patients and methods

### Patients

Adult (≥20 years old) patients with initial or recurrent (up to first recurrence) indolent B cell lymphoma [FL, MALT lymphoma, nodal and splenic marginal zone B cell lymphomas (NMZB, SMZB), lymphoplasmacytic lymphoma (LPL) ≈ Waldenstrom macroglobulinemia (WM), hairy cell leukemia (HCL), etc.] or MCL, who were pathologically diagnosed with Ann Arbor stages II–IV and confirmed to be CD20 positive with pathology or flow cytometry, were selected. Exclusion criteria were as follows: CD20 negative, severe infection, prior history of receiving similar chemotherapy for treating malignant lymphoma, HIV positive, HTLV1 positive, HBV-Ag positive, high titer of HBc-Ab, scheduled to undergo autologous or allogeneic hematopoietic stem cell transplantation and determined as unsuitable for the study by a physician. A total of 33 patients who received care between January 2008 and August 2011 at one of the four centers were enrolled. The disease types were as follows: FL (*n* = 21), NMZB (*n* = 3), MCL (*n* = 3), SMZB (*n* = 2), HCL (*n* = 1), WM (*n* = 1), and LPL (*n* = 2). There were 17 men and 16 women, with a median age of 65 years (range 45–81 years). Nine patients had previously undergone treatment (Table 1). In addition, all patients enrolled in the study gave written consent themselves. This study was approved by the IRB of each center.

**Table 1:** Patient characteristics

	No. (%)
Patients	33
Sex	
Male	17 (52)
Female	16 (48)
Age, years	
Median	65
Range	45–81
Histopathology	
Follicular lymphoma	21 (64)
Nodal marginal zone lymphoma	3 (9)
Mantle cell lymphoma	3 (9)
Splenic marginal zone lymphoma	2 (6)
Hairy cell leukemia	1 (3)
Waldenstrom macroglobulinemia	1 (3)
Lymphoplasmacytic lymphoma	2 (6)
Prior therapy	9 (27)
Stage	
1	0 (0)
2	8 (24)
3	10 (30)
4	15 (46)
B symptoms	2 (6)

B symptoms: fever, weight loss, night sweats; prior therapy: R-CHOP, rituximab, splenectomy, plasma exchange, prednisolone, VP16, auto-peripheral blood stem cell transplantation

Stage I was not included

## Methods

Patients received four 21-day cycles of rituximab 375 mg/m^2^ (day 1), cladribine 0.10 mg/kg (days 1–3), mitoxantrone 8 mg/m^2^ (day 1), and dexamethasone 8 mg/body (days 1–3) and additionally received four times of rituximab at 4-week intervals (Table 2). For prophylaxis, sulfamethoxazole/trimethoprim combination was given at a dose of 800 mg of sulfamethoxazole/day twice a week to prevent pneumocystis infection, and an antifungal agent was given from the start of the treatment to 2 months after its completion to prevent mycosis. Preventative administration of common antibiotics was not specified. Vaccinations for influenza and other diseases were avoided from the start of the treatment to 3 months after its completion.

**Table 2:** R-CMD regimen

Drugs	Dose	Day 1	Day 2	Day 3
Rituximab	375 mg/m^2^	↓		
Cladribine	0.10 mg/kg	↓	↓	↓
Mitoxantrone	8 mg/m^2^	↓		
Dexamethasone	8 mg/body	↓	↓	↓

R-CMD, rituximab plus cladribine, mitoxantrone, and dexamethasone; R-CMD regimen: four 3-week R-CMD regimen + four 4-week rituximab monotherapy regimen

The complete response (CR) rate and adverse events were determined in accordance with the standardized response criteria for non-Hodgkin’s lymphoma from an international workshop report by Cheson et al. [10] and CTCAGv4.0 (UMIN trial ID: 1341), respectively. The primary end point was the CR rate, and the secondary end points were the frequency of adverse events, overall survival (OS), and overall response rate (ORR). Patients were followed until death or 10 years after treatment completion, whichever occurred first. OS curves were estimated using the Kaplan–Meier method.

## Results

### Response

A complete response/unconfirmed complete response (CR/CRu) was achieved in 26 out of 33 patients (79 %), and a partial response (PR) was achieved in six patients (18 %). The ORR was 97 %, with one patient whose condition progressed (3 %). Moreover, excluding patients with MCL, 24 patients (80 %) had CR, five patients (17 %) had PR, and one patient (3 %) had disease progression. There were only three patients with MCL, and two of these patients achieved CR/Cru, whereas the remaining one patient achieved PR (Table 3). In newly diagnosed patients alone, CR/CRu, PR, and progressive disease (PD) were observed in 83, 16, and 0 %, respectively. In addition, recurrence was observed in one patient with FL, two patients with MCL, and one patient with LPL. CR/CRu was 67 %, PR was 22 %, and PD was 11 % in patients with previous treatment history.

**Table 3:** Response

	All	Non-MCL	MCL
	*N* = 33	*N* = 30	*N* = 3
CR/Cru (%)	26 (79)	24 (80)	2 (67)
PR (%)	6 (18)	5 (17)	1 (33)
SD (%)	0 (0)	0 (0)	0 (0)
PD (%)	1 (3)	1 (3)	0 (0)
ORR (%)	32 (97)	29 (97)	3 (100)

*CR* complete response, *CRu* unconfirmed CR, *PR* partial response, *SD* stable disease, *PD* progressive disease, *ORR* overall response rate, which includes CR + CRu + PR, *All* All enrolled patients, *MCL* mantle cell lymphoma

### Disease-free survival and OS

**Fig. 1:**
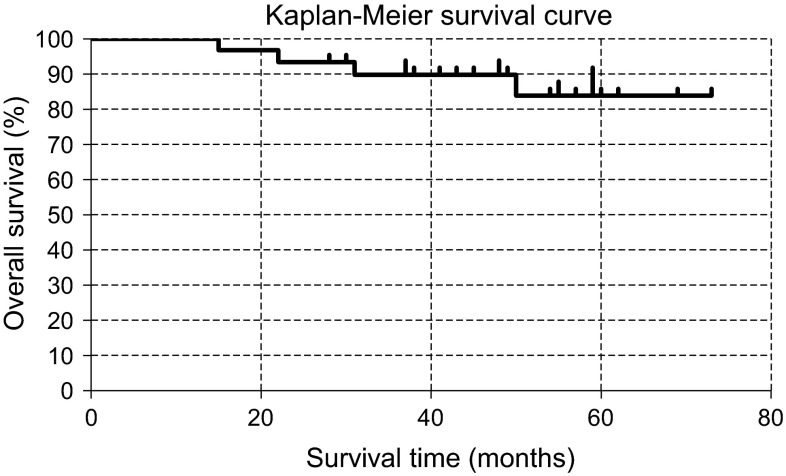
Overall survival for all patients after R-CMD therapy

At the present time, recurrence has been observed in 12 % of the patients (1 patient with FL, 2 patients with MCL, and 1 patient with LPL). No other recurrences have been observed, and the maximum disease-free survival thus far has been 5 years and 4 months. With a median follow-up of 4 years, the cumulative survival rate was 84 % with four deaths at the 73-month time point (Fig. 1). These deaths were due to progression in lymphoma combined with pancreatic cancer that developed later (*n* = 1), metastatic liver cancer (*n* = 1), cirrhosis type C (*n* = 1), or pneumocystis pneumonia (*n* = 1).

### Toxicity

Grade 3 or greater hematological toxicity was observed in 30 out of 33 patients. Specifically, ≥grade 3 leukopenia,neutropenia,lymphopenia, and thrombocytopenia were observed in 73, 76, 71, and 6 % of the patients, respectively. None of the patients exhibited ≥grade 3 anemia (Table 4). Advanced neutropenia and lymphopenia were observed, but all cases were transient. Non-hematological toxicity included grade 2 constipation in 27 % and alopecia in 6 % of the patients. Additionally, five patients (15 %) developed an infection (one patient each with sepsis + herpes labialis, pyothorax + bacterial fasciitis, urinary tract infection, herpes zoster, and pneumocystis pneumonia). Although the patient with pneumocystis pneumonia died without showing any improvements, other infections did not become serious and improved with treatment. However, treatment continuation was determined to be difficult in the patients who developed pyothorax + bacterial fasciitis and urinary tract infection, and these patients subsequently discontinued the regimen. Although pancreatic cancer developed in one patient after the completion of treatment, secondary cancers such as myelodysplastic syndrome or leukemia have not been confirmed at the present time.

**Table 4:** Adverse events

	Grade 1	Grade 2	Grade 3	Grade 4
	*N* (%)	*N* (%)	*N* (%)	*N* (%)
Hematological				
Leukopenia	1 (3)	8 (24)	19 (58)	5 (15)
Neutropenia	1 (3)	6 (18)	15 (46)	10 (30)
Lymphopenia	1 (4)	5 (18)	16 (57)	4 (14)
Anemia	16 (49)	8 (24)	0 (0)	0 (0)
Thrombocytopenia	8 (24)	3 (9)	2 (6)	0 (0)
Non-hematological				
Constipation	10 (30)	9 (27)	0	0
Alopecia	9 (27)	2 (6)	–	–
Infections	2 (6)	1 (3)	12	0
Infusion-related reaction	0	1 (3)	0	0
Rash	0	2 (6)	0	0
Other	2 (6)			

Other non-hematological toxicities included two patients with hand stiffness

One patient died due to pneumocystis pneumonia (grade 5)

Two patients discontinued treatment due to adverse events (one patient with pyothorax + necrotizing bacterial fasciitis and one patient with urinary tract infection)

## Discussion

Due to the slow disease progression of indolent B cell lymphomas, which primarily consist of FL, it is rare for sudden exacerbations to occur after its onset. Nonetheless, standardized treatments have yet to be established for this disease. Although temporary effects of the conventional CHOP regimen are observed in indolent B cell lymphoma, this particular therapy does not achieve cure, and the long-term prognosis is known to be rather worse than that of aggressive lymphoma. In addition, standardized treatments for MCL, a disease with poor prognosis that most commonly affects the elderly, have yet to be established as well. With the recent introduction of bendamustine and ibritumomab tiuxetan, an improvement in survival and prognosis is anticipated; however, it is unknown whether or not either of these drugs alone can achieve a complete cure in a monotherapy regimen [11–13]. Rummel et al. [14] conducted a phase III multicenter randomized controlled trial to compare the efficacy of the bendamustine plus rituximab (RB) regimen versus the R-CHOP regimen in patients with indolent B cell lymphoma and MCL and reported that the RB regimen may be more beneficial. In this trial, progression-free survival (PFS) was significantly better in the RB group compared to the R-CHOP group in the overall study population inclusive of all disease types (*p* < 0.0001). In the analysis by disease type, the authors found that PFS was significantly longer in FL, MCL, and primary WM, but a significant difference was not observed in marginal-cell lymphoma. There was no significant difference in ORR (93 % in the RB group and 91 % in the R-CHOP group); however, the CR rate was 40 % in the RB group and 30 % in the R-CHOP group, indicating a superiority of the RB regimen (*p* = 0.021). OS was not significantly different between the two groups. Sinha et al. conducted a phase I trial of the combined regimen with bortezomib and R-CHOP in previously untreated patients with indolent B cell lymphoma [15] and found favorable outcomes with an ORR of 100 %, CR of 68 %, and 3-year PFS of 89.5 %. Furthermore, Fowler et al. [16] reported the results a phase II trial of the combination regimen of bortezomib, bendamustine, and rituximab (VERTICAL trial) in 73 patients with relapsed or refractory FL and concluded that ORR was 88 %, CR was 53 %, and the median PFS was 14.9 months. Leonard et al. [17] evaluated the combination regimen of lenalidomide and rituximab in recurrent and refractory FL (CALGB trial) and reported that the ORR was 75 % with a CR of 32 % and event-free survival of 24 months.

In the present study, 26 out of 33 patients achieved complete remission (CR rate, 79 %). At the present time, with the exception of the recurrence that occurred in 12 % of the patients (1 with FL, 2 with MCL, and 1 with LPL) and PD in 3 % of the patients (1 with FL), recurrence has not been observed for a maximum of 5 years and 4 months. The cumulative survival rate at the 73-month time point was 84 %, indicating results that are comparable to similar regimens that have been reported previously (Table 5) [1, 18–22]. Therefore, we consider that R-CMD therapy is a relatively convenient and effective treatment method for indolent B cell lymphoma. In addition, although ≥grade 3 leukopenia, neutropenia, and lymphopenia were observed at high frequencies, all cases were transient and none of them worsened. Serious non-hematological toxicities were rare in patients who received treatment for the first time, and alopecia, which is highly common in R-CHOP, was also rarely observed. In addition, although a combination regimen with multiple drugs that include cladribine is known to cause adverse events at high frequencies in recurrent and refractory lymphomas, toxicity is considered to be within the tolerable range in newly diagnosed patients. Nonetheless, thorough infection prevention is necessary to suppress the number and function of both B cells and T cells.

**Table 5:** Similar clinical trials

Study	*n*	Regimen	ORR (%)	CR (%)	Response time	OS (years)
Apostolia et al. [1] (untreated indolent NHL maintenance therapy+)	73	FND	97	79	41 % (FFS)	84 (5)
Velasquez et al. [18] (untreated indolent NHL)	78	FM	94	44	38 % (PFS)	88 (4)
McLaughlin et al. [19] (untreated SLL or follicular lymphoma)	149	FND-R	100	92	77 % (FFS)	95 (3)
Bordonaro et al. [20] (newly diagnosed indolent NHL)	18	FND	94	72	52 % (PFS)	67 (2)
Montoto et al. [21] (untreated follicular lymphoma)	120	FCM	94	83	58 % (PFS)	89 %
Tomasz et al. [22] (relapse or refractory indolent NHL)	28	FPD-R	89	63	74 % (TTP)	92 (3)
This study, 2013 (untreated or first relapse indolent NHL)	33	R-CMD	97	78	–	84 (4)

*ORR* overall response rate; *CR* complete response; *PFS* progression-free survival; *OS* overall survival; *FFS* failure-free survival; *TTP* time to tumor progression; *FND* fludarabine, mitoxantrone, and dexamethasone; *FM* fludarabine and mitoxantrone; *FND*-*R* FND plus rituximab; *FCM* fludarabine, cyclophosphamide, and mitoxantrone; *FPD*-*R* pixantrone, fludarabine, and dexamethasone plus rituximab; *R*-*CMD* rituximab plus cladribine, mitoxantrone, and dexamethasone; *SLL* small lymphocytic lymphoma; *NHL* non-Hodgkin lymphoma

## Conclusion

R-CMD therapy, a regimen with rituximab added to the FMD regimen in which fludarabine is switched to cladribine, showed effects that were comparable to FMD and a favorable complete remission induction rate. However, the observation time is still short, and further follow-up is necessary in the future to assess long-term prognosis.
